# Public knowledge, stigma, and social acceptance toward mental illness in the Gulf region: a cross-sectional survey

**DOI:** 10.3389/fpsyt.2026.1860027

**Published:** 2026-06-30

**Authors:** Hamad Alhamad, Najla Alsiri, Mohammad Alshehab, Mohammad Alenezi, Sheikha Alqallaf

**Affiliations:** 1 Occupational Therapy Department, Faculty of Allied Health Sciences, Kuwait University, Health Science Center, Kuwait City, Kuwait; 2 Physical Therapy Department, Faculty of Allied Health Sciences, Kuwait University, Health Science Center, Kuwait City, Kuwait; 3 Alrazi Orthopedic Hospital, Physical Therapy Department, Capital Governance, Kuwait City, Kuwait; 4 Occupational Therapy Department, Ministry of Health, Kuwait City, Kuwait; 5 Registrar, Internal Medicine Department, Farwaniya Hospital, Ministry of Health of Kuwait, Kuwait

**Keywords:** mental illness, public stigma, mental-health literacy, social distance, Gulf Cooperation Council, cross-sectional survey, Arabic validation

## Abstract

**Background:**

Mental illness stigma undermines help-seeking, treatment engagement, and social inclusion globally. Although the Gulf Cooperation Council (GCC) region has undergone rapid social and health-system modernization, few studies have simultaneously assessed public knowledge, attitudes, and intended behaviour toward people with mental illness using validated instruments.

**Objective:**

To examine mental-health knowledge, public stigma, and social acceptance among adults in GCC countries using the Mental Health Knowledge Schedule (MAKS), the Reported and Intended Behaviour Scale (RIBS), and the 40-item Community Attitudes toward the Mentally Ill (CAMI) scale.

**Methods:**

An Arabic-language online survey was completed by 1, 557 adults (84.1% female; mean age = 26.2 years, *SD* = 10.6; 80.5% from Kuwait). Descriptive statistics, bivariate analyses, and simultaneous-entry multiple linear regression were conducted. Internal consistency was evaluated using Cronbach’s α and McDonald’s ω, supplemented by item-total correlations and confirmatory factor analysis (CFA) of the CAMI.

**Results:**

Participants reported moderate mental-health knowledge (MAKS *M* = 41.6, *SD* = 5.9), moderately positive attitudes (CAMI *M* = 135.2, *SD* = 16.2), and moderate behavioural willingness (RIBS *M* = 13.1, *SD* = 3.2). Knowledge and attitudes were moderately correlated (*r* = .341, *p* <.001), but the association between attitudes and intended behaviour was weaker (*r* = .209, *p* <.001). In regression, social restrictiveness was the strongest predictor of behavioural engagement (β = .374, *p* <.001), followed by male sex (β = .098) and MAKS total (β = −.103). A confirmatory factor analysis indicated poor fit of the original four-factor CAMI structure (CFI = .673, RMSEA = .141), supporting cautious subscale-level interpretation. A supplementary regression using the more reliable CAMI total score confirmed the primary pattern. Sensitivity analyses following removal of implausible age entries and restricting to Kuwait-only participants produced substantively identical findings.

**Conclusion:**

These findings, drawn from a predominantly Kuwaiti, female, and university-educated convenience sample, indicate that respondents endorsed sympathetic and recovery-oriented principles yet showed substantial hesitation regarding close social contact. The disconnect between attitudes and behavioural willingness highlights the need for culturally grounded, contact-based anti-stigma interventions that target social distance specifically, while requiring replication in more representative GCC populations.

## 1 Introduction

### 1.1 The nature and consequences of mental illness stigma

Mental illness stigma is widely recognised as a multidimensional public-health and human-rights challenge that compromises knowledge, attitudes, behaviour, access to services, recovery, and social inclusion (1–4. Goffman (5) first conceptualized stigma as a socially discrediting attribute that reduces an individual from a whole to a tainted person. Building on this foundation, Link and Phelan (6) proposed a comprehensive model in which stigma emerges through the convergence of labelling, stereotyping, separation, status loss, and discrimination under conditions of power asymmetry. Corrigan and colleagues further distinguished public stigma—stereotypes, prejudice, and discrimination enacted by the general population—from self-stigma, in which individuals internalize negative societal beliefs about their condition (1, 7). Structural stigma extends these processes into institutional practices, policies, legislation, and service delivery, thereby shaping life chances and access to care at the population level (2, 3, 8).

Within the social-cognitive model of stigma, Corrigan (1) proposed that public stigma operates through three sequential components: stereotypes (cognitive representations about a group), prejudice (endorsement of stereotypes accompanied by negative emotional reactions), and discrimination (behavioural responses such as avoidance or exclusion). This tripartite model has been influential in stigma research because it highlights that knowledge, attitudes, and behaviour are related but separable domains, and that change in one does not guarantee change in another (9). Population-level surveys conducted over several decades have consistently demonstrated that perceptions of dangerousness, unpredictability, and desire for social distance remain common public responses to mental illness across diverse cultural settings (10–13).

The consequences of stigma for individuals living with mental illness are substantial and well documented. A systematic review by Clement et al. (14) concluded that stigma exerts a small-to-moderate negative effect on help-seeking across both quantitative and qualitative studies. Schnyder et al. (15) found that personal stigma, self-stigma, and perceived public stigma are each independently associated with reduced active help-seeking. Beyond treatment barriers, stigma undermines employment opportunities, housing stability, social relationships, self-esteem, and overall quality of life (2, 16–18). The WHO (4) has identified stigma reduction as a global priority for mental-health system transformation.

### 1.2 Mental health literacy and its relationship to stigma

Understanding how stigma operates requires attention not only to attitudes but also to knowledge. Mental health literacy (MHL)—originally defined as knowledge and beliefs about mental disorders that aid their recognition, management, or prevention (19)—has become central to stigma theory because low literacy can normalise avoidance, discourage professional consultation, and reinforce negative stereotypes (20, 21). Later formulations expanded the concept to include disorder recognition, knowledge of risk factors, awareness of available professional help, and attitudes that facilitate appropriate help-seeking (21, 23); notably, population-level mental health literacy can shift over time in response to public awareness initiatives (22). A global review by Gulliver et al. (24) found that poor MHL was a key barrier to help-seeking among young people, and Furnham and Swami (25) documented substantial cross-cultural variation in public recognition of mental disorders.

However, improvements in literacy do not necessarily translate into equivalent improvements in social acceptance or behavioural inclusion. Schomerus et al. (12) demonstrated in a meta-analysis of temporal trends that while public understanding of mental illness improved in several high-income countries, social acceptance did not keep pace with knowledge gains. This dissociation has led scholars to emphasize that literacy is necessary but insufficient; public understanding must be accompanied by willingness to include people with mental illness in everyday social roles (3, 14, 26).

### 1.3 Social distance and the attitude–behaviour gap

Social distance—the degree to which individuals seek to avoid close interpersonal contact with members of a stigmatized group—remains one of stigma’s clearest behavioural manifestations (27, 28). Research has consistently shown that people may endorse general sympathy or abstract rights-based principles while simultaneously hesitating to live with, work with, or befriend someone with a mental-health condition (2, 10, 29, 30). Pescosolido et al. (30) found that American public willingness to interact with people with mental illness did not increase between 1996 and 2006 despite improved etiological understanding, and Evans-Lacko et al. (26) documented a persistent gap between attitudes and behavioural intentions in a large English population survey. These findings underscore the importance of measuring not only what people believe about mental illness but also what they report being willing to do in relation to people with mental illness.

### 1.4 Stigma reduction through social contact

Among the most consistently supported approaches to stigma reduction is meaningful social contact with people who have lived experience of mental illness. Corrigan et al. (31) demonstrated in a meta-analysis that contact-based strategies outperformed both protest-based and education-only approaches in reducing public stigma. The Lancet Commission on ending stigma and discrimination likewise identified social contact, co-production with people with lived experience, and rights-based action as essential components of sustainable stigma reduction (3). More recent meta-analyses have reinforced that anti-stigma interventions can improve knowledge and attitudes, although behavioural effects tend to be smaller and long-term sustainability remains uncertain (32–39). These findings collectively suggest that behavioural change requires more than information provision; it requires facilitation of meaningful interpersonal experiences that challenge stereotypes and reduce perceived social costs of contact.

### 1.5 Mental illness stigma in the Arab and GCC context

Region-specific data remain limited but increasingly suggest that mental illness stigma in Arab societies is substantial and shaped by distinctive sociocultural forces. In the GCC countries (Bahrain, Kuwait, Oman, Qatar, Saudi Arabia, and the United Arab Emirates) and the wider Arab region, stigma is influenced not only by knowledge gaps but also by family honour norms (*ird* and *sharaf*), concerns about social reputation and marriageability, religious and moral interpretations of psychological distress (including attributions to spiritual or supernatural causes), collectivist values emphasizing family cohesion and privacy, and strong expectations around nondisclosure of family difficulties (40–45).

Elyamani et al. (43) conducted the first systematic review of MHL in GCC countries and identified sparse, heterogeneous evidence with significant gaps in public knowledge, disorder recognition, and culturally relevant intervention research. Fekih-Romdhane et al. (44) found that religiosity moderated the relationship between stigma and help-seeking attitudes across 16 Arab countries, with religiosity exerting a protective effect in some contexts but reinforcing stigma in others. Studies from Saudi Arabia have documented widespread public endorsement of supernatural causal models alongside growing biomedical understanding (46, 47), and Aljuhnie et al. (48) reported substantial stigma toward help-seeking in a large Makkah sample. In Oman, Al-Hashemi et al. (49) found that primary healthcare physicians—despite clinical training—also held stigmatizing views, suggesting that stigma penetrates even professional populations. Al Omari et al. (50) documented public and self-stigma among university students across 11 Arabic-speaking countries, and BinDhim et al. (51) found that Saudi public attitudes were highly variable by age, education, and urban versus rural residence. In Lebanon, Abi Doumit et al. (40) found that the public frequently endorsed stereotypes about dangerousness and incompetence while simultaneously expressing sympathy. Qualitative research has further documented that Arab families may refuse psychotherapy to protect the reputation of the family (42), and that concerns about marriageability and community standing serve as primary barriers to mental-health engagement in Gulf Arab states (52, 53).

Despite these contributions, relatively few GCC studies have assessed knowledge, attitudes, and intended behaviour together using validated, multi-domain stigma measures, limiting the evidence base for targeted intervention design. Most existing studies have used single instruments or unstandardized items, making cross-study comparison difficult and obscuring the specific dimensions of stigma that are most consequential for behavioural outcomes in the region (43, 45).

### 1.6 Instruments and study rationale

To address this gap, the present study used three established instruments that together capture distinct but related stigma domains. The Mental Health Knowledge Schedule (MAKS; 54) assesses stigma-related MHL and is widely used in population monitoring and anti-stigma programme evaluation (55). The Reported and Intended Behaviour Scale (RIBS; 29) measures reported contact and intended future behaviour, providing a behavioural index of social distance and inclusion. The Community Attitudes toward the Mentally Ill scale (CAMI; 56) remains one of the most frequently used tools for assessing community attitudes, with a recent systematic review of its psychometric properties supporting its broad utility while noting variability in subscale performance across cultural settings (57). Arabic validation work has supported the applicability of MAKS and RIBS in Arab populations, although psychometric performance varies by adaptation and population (58).

### 1.7 Study aims

Drawing on the social-cognitive model of public stigma (1, 9), the present study addressed three aims: (a) to describe levels of mental-health knowledge, public attitudes, and intended social behaviour toward people with mental illness in a GCC sample; (b) to examine the interrelationships among these three domains; and (c) to identify demographic and attitudinal predictors of behavioural willingness toward social inclusion. We hypothesized that knowledge would be positively associated with attitudes, but that the relationship between attitudes and behavioural willingness would be weaker, consistent with the established attitude–behaviour gap in stigma research (12, 26).

## 2 Methods

### 2.1 Participants and procedure

An Arabic-language online survey was administered between 18 February and 19 March 2026 using Google Forms. The survey link was distributed through social media platforms (including Twitter/X, Instagram, and WhatsApp groups) and university networks. Eligible participants were adults aged 18 years or older, residing in a GCC country, and able to read Arabic. Although the original study protocol was developed around Kuwait-focused recruitment, the realized sample included respondents from all six GCC countries and therefore represents a non-probability community survey of Arabic-speaking young adults across GCC states rather than a representative national or regional sample. The demographic composition—predominantly female (84.1%), university-educated (79.0%), and Kuwait-based (80.5%)—is consistent with the profile commonly observed in comparable Arabic-language online stigma surveys (58, 59) but should be considered when evaluating the scope of the findings.

A total of 1, 557 participants completed the survey in full. *Post-hoc* inspection of self-reported age data revealed 97 cases with age values below 18 years, including two implausible entries below age 10, likely reflecting typographical errors. These cases were retained in primary analyses, with a sensitivity analysis excluding all under-18 entries reported to confirm robustness (see Results). For the regression analysis with 19 predictors, the achieved sample exceeds conventional recommendations (60) and provides adequate statistical power to detect small-to-medium effects.

### 2.2 Ethical considerations

This study was conducted in accordance with the ethical principles of the Declaration of Helsinki. Ethical approval was granted by the Kuwait University Health Sciences Centre Ethics Committee (Ref: XX). Participants received an information sheet outlining the study’s goals, the voluntary nature of participation, and their right to withdraw at any time without adverse consequences. Online informed consent was obtained from all participants prior to participation. Anonymity and confidentiality were maintained throughout, and only aggregated data are reported.

### 2.3 Instruments

#### 2.3.1 Mental health knowledge schedule

The MAKS contains 12 items assessing stigma-related MHL: six general knowledge items (C1–C6) rated on a 6-point scale and six condition-recognition items (C14–C19) rated on a 5-point scale (54). In the Arabic response format, “don’t know” was presented as a separate option from neutral; to preserve comparability with published scoring norms—both MAKS and RIBS were originally developed with 5-point response scales where the midpoint subsumes uncertainty (29, 54)—”don’t know” and neutral responses were collapsed during score derivation, consistent with the scoring approach used in the Arabic validation by Ben Amor et al. (58). We acknowledge that these response categories may reflect distinct psychological states and that sensitivity analyses examining the impact of this collapsing decision would be a valuable direction for future psychometric research with Arabic stigma instruments. Items were coded so that higher total scores indicate greater knowledge (possible range: 12–60).

#### 2.3.2 Reported and intended behaviour scale

The RIBS includes eight items (29). Items 1–4 capture reported prior or current contact with people who have mental illness (yes/no/don’t know) and are treated descriptively. Items 5–8 assess intended future social behaviour on a Likert-type scale; “don’t know” and neutral were collapsed as described above, yielding a summed behavioural willingness score with higher scores indicating greater willingness for social inclusion (possible range: 4–20).

#### 2.3.3 Community attitudes toward the mentally Ill

The original 40-item CAMI (56) was used. Items are rated on a 5-point Likert-type scale across four interleaved subscales of 10 items each: Authoritarianism (items 1, 5, 9, 13, 17, 21, 25, 29, 33, 37), Benevolence (items 2, 6, 10, 14, 18, 22, 26, 30, 34, 38), Social Restrictiveness (items 3, 7, 11, 15, 19, 23, 27, 31, 35, 39), and Community Mental Health Ideology (CMHI; items 4, 8, 12, 16, 20, 24, 28, 32, 36, 40). Positively worded items were reverse-coded so that higher subscale and total scores indicate less stigmatizing and more accepting attitudes (subscale range: 10–50; total range: 40–200).

#### 2.3.4 Translation and adaptation

The Arabic versions of MAKS, RIBS, and the 40-item CAMI used in the present study were obtained from the supplementary material published by Abi Doumit et al. (40), who conducted a forward–back translation of all three instruments for use in a national Lebanese sample (*N* = 2, 289). In their translation process, the English versions were translated into Arabic by a mental-health specialist, then back-translated into English by an independent specialist. An expert committee comprising healthcare professionals and a language professional reviewed the Arabic versions to identify and resolve discrepancies, and the forward–back translation process was repeated until all ambiguities were resolved (40). Internal consistency in that study was adequate for the CAMI total (α = .876; subscale range:.555–.804), MAKS (α = .749), and RIBS (α = .766). No further modifications were made to the item wording for the present study. In the Arabic response format for MAKS and RIBS, “don’t know” was presented as a separate option from the neutral midpoint. To preserve comparability with published scoring norms—both MAKS and RIBS were originally developed with 5-point response scales where the midpoint subsumes uncertainty (29, 54)—”don’t know” and neutral responses were collapsed during score derivation, consistent with the scoring approach used in the Arabic validation by Ben Amor et al. (58). We acknowledge that these response categories may reflect distinct psychological states, and sensitivity analyses examining the impact of this collapsing decision would be a valuable direction for future psychometric research with Arabic stigma instruments.

### 2.4 Data analysis

All analyses were conducted using IBM SPSS Version 31 and Jamovi Version 2.6 (61), with confirmatory factor analysis performed via the SEMLj module (62) using the lavaan R package (63). Descriptive statistics were used to summarize participant characteristics and scale scores. Internal consistency was assessed using both Cronbach’s α and McDonald’s ω (64). Corrected item-total correlations were examined for subscales with α below.50 to identify poorly performing items. A confirmatory factor analysis (CFA) of the 40-item CAMI was conducted to evaluate the original four-factor structure (56) in the present Arabic-speaking GCC sample. The model was estimated using diagonally weighted least squares (DWLS), appropriate for ordinal Likert-scale data. Model fit was evaluated using the Comparative Fit Index (CFI), Tucker-Lewis Index (TLI), Root Mean Square Error of Approximation (RMSEA), and Standardized Root Mean Square Residual (SRMR).

Normality of score distributions was examined using the Kolmogorov–Smirnov and Shapiro–Wilk tests; both indicated statistically significant departures from normality (*p* = .001). However, with *N* > 1, 500, the sampling distributions of means are approximately normal by the central limit theorem, and parametric tests are robust to non-normality under these conditions (60, 65). Parametric analyses were therefore retained.

Bivariate associations were examined using Pearson correlations (for continuous variables), independent-samples *t* tests (for sex), and one-way ANOVA with Bonferroni-corrected *post-hoc* pairwise comparisons (for country, education, and occupation). Effect sizes are reported as Cohen’s *d* for *t* tests and partial η² for ANOVAs, following APA reporting standards. A simultaneous-entry multiple linear regression was conducted to predict RIBS total score from the four CAMI subscale scores, MAKS total, age, sex, educational level, country (dummy-coded with Bahrain as the reference category), and occupation (dummy-coded with employee as the reference category). A supplementary regression using the CAMI total score as the sole attitudinal predictor was conducted to evaluate model robustness given the suboptimal subscale reliabilities, following the recommendation of Sanabria-Mazo et al. (57) that the CAMI global score demonstrates more consistent reliability than individual subscales.

Regression assumptions were evaluated through comprehensive diagnostics. Independence of residuals was assessed using the Durbin-Watson statistic. Collinearity was assessed using variance inflation factors (VIF) and tolerance values; all VIF values were below 1.70 and tolerance above 0.59, well within conventional bounds (66). Homoscedasticity and linearity were assessed via visual inspection of residual plots. Influential observations were identified via Cook’s distance. Residual normality was examined via Q-Q plot inspection. The overall effect size was quantified using Cohen’s *f*² (67).

Following reviewer feedback and additional quality screening, implausible age values were identified and treated as missing. The corrected age distribution demonstrated a mean of 26.65 years (*SD* = 10.53; range = 17–68). Regression analyses were subsequently recomputed using the cleaned dataset (*N* = 1, 485). A further sensitivity analysis restricted to Kuwait-only respondents was conducted to verify that primary findings were not driven by between-country compositional effects. Statistical significance was set at *p* <.05 (two-tailed).

## 3 Results

### 3.1 Sample characteristics

A total of 1, 557 participants completed the survey. The mean age was 26.2 years (*SD* = 10.6; range = 2–68), and 84.1% identified as female. Most participants resided in Kuwait (80.5%), followed by Saudi Arabia (11.5%); participants from Bahrain (3.0%), the UAE (2.6%), Qatar (1.3%), and Oman (1.1%) comprised the remaining 8.0%. The majority reported undergraduate-level education (79.0%), and the largest occupational group was university students (59.7%), followed by employees (25.2%). Full demographic characteristics are presented in **Table 1**.

**Table 1:** Sociodemographic characteristics of the participants (N = 1, 557).

Variable	Category	n (%)/M (SD)
Age (years)		26.2 (10.6)
Sex	Male	248 (15.9)
	Female	1, 309 (84.1)
Country	Kuwait	1, 254 (80.5)
	Saudi Arabia	179 (11.5)
	Bahrain	47 (3.0)
	Qatar	20 (1.3)
	Oman	17 (1.1)
	UAE	40 (2.6)
Education	Postgraduate	83 (5.3)
	Undergraduate	1, 230 (79.0)
	High school	197 (12.7)
	Middle school	44 (2.8)
	Primary	3 (0.2)
Occupation	Employee	392 (25.2)
	Unemployed	92 (5.9)
	University student	930 (59.7)
	Retired	70 (4.5)
	Unable to work	10 (0.6)
	Self-employed	37 (2.4)
	Health-related sector	26 (1.7)

Values are presented as frequency (percentage) unless otherwise indicated.

### 3.2 Internal consistency and psychometric analysis

Internal consistency for the CAMI total scale was good (α = .836, ω = .816). At the subscale level, Authoritarianism (α = .651, ω = .667) and Benevolence (α = .626, ω = .677) demonstrated moderate reliability, while CMHI showed lower but acceptable consistency (α = .573, ω = .632). Social Restrictiveness demonstrated poor reliability (α = .403, ω = .482), suggesting potential cultural or conceptual variability in item interpretation within the study population. These findings are broadly consistent with prior literature documenting variable CAMI subscale performance across translated and culturally adapted versions; Sanabria-Mazo et al. (57) reported subscale alphas ranging from.27 to.68 across 15 international CAMI studies (*N* = 10, 841), and the Persian CAMI validation reported Benevolence α = .49 (68). Internal consistency for RIBS was good (α = .864, ω = .807). For MAKS, total 12-item reliability was α = .653 (ω = .530); however, the two component sections performed differently, with the general knowledge items (C1–C6; α = .720) showing acceptable reliability and the condition-recognition items (C14–C19; α = .805) showing good reliability. The suboptimal reliability of CAMI subscales—particularly Social Restrictiveness—and the MAKS total score should be borne in mind when interpreting findings at the subscale level; the direction of bias from measurement error is toward attenuation of regression coefficients (69), meaning that significant effects are likely conservative estimates.

Item-level analyses for the Social Restrictiveness subscale indicated that the reduced reliability was not attributed to a single problematic item, but rather reflected modest inter-item correlations across several items. No single item deletion substantially improved reliability. Three items showed particularly low corrected item-total correlations: Item 11 (*r*_it_ = −.056), Item 19 (*r*_it_ = −.022), and Item 27 (*r*_it_ = −.013), while the remaining items ranged from.119 to.335 (see **Supplementary Table S1**). These findings suggest that the low internal consistency reflects broader measurement instability in the Arabic adaptation rather than a single problematic item.

### 3.3 Confirmatory factor analysis of the Arabic CAMI

A confirmatory factor analysis was conducted to evaluate the four-factor structure of the Arabic CAMI within the present sample. Using diagonally weighted least squares (DWLS) estimation appropriate for ordinal Likert-scale data, the original four-factor model (Authoritarianism, Benevolence, Social Restrictiveness, and CMHI) demonstrated poor fit indices: CFI = .673, TLI = .653, RMSEA = .141, SRMR = .154. These values fall below conventional thresholds for acceptable fit (CFI/TLI >.90; RMSEA <.08; SRMR <.08; 70). Several Social Restrictiveness items demonstrated weak or inconsistent factor loadings, consistent with the low internal consistency observed for this subscale. These findings indicate limited structural fit of the original CAMI factor structure in this GCC Arabic sample and suggest potential cultural or contextual instability in the measurement properties of specific CAMI dimensions. This is consistent with the Sanabria-Mazo et al. (57) systematic review finding that neither the three- nor four-factor CAMI structure replicates consistently across translations internationally. These findings support the use of the CAMI total score as the primary measure of community attitudes, with subscale scores treated as exploratory.

### 3.4 Descriptive statistics

The mean CAMI total score was 135.2 (*SD* = 16.2; possible range: 40–200; midpoint = 120), indicating a moderately positive overall attitude toward people with mental illness. At the subscale level (each possible range: 10–50; midpoint = 30), Authoritarianism (*M* = 32.6, *SD* = 5.3), Benevolence (*M* = 34.8, *SD* = 5.4), Social Restrictiveness (*M* = 32.3, *SD* = 4.2), and CMHI (*M* = 35.6, *SD* = 5.1) were all above the subscale midpoint, suggesting that respondents leaned toward less stigmatizing views across all domains while still endorsing some controlling and socially restrictive beliefs. The mean MAKS score was 41.6 (*SD* = 5.9; possible range: 12–60; midpoint = 36), indicating moderate-to-good mental-health knowledge. The mean RIBS score was 13.1 (*SD* = 3.2; possible range: 4–20; midpoint = 12), indicating moderate behavioural willingness. Full descriptive statistics are presented in **Table 2**.

**Table 2:** Descriptive statistics, internal consistency, and score ranges (N = 1, 557).

Measure	Range	Midpoint	M (SD)	Min	Max	α	ω
CAMI Total	40–200	120	135.2 (16.2)	84	191	.836	.816
Authoritarianism	10–50	30	32.6 (5.3)	15	50	.651	.667
Benevolence	10–50	30	34.8 (5.4)	18	50	.626	.677
Social Restrict.	10–50	30	32.3 (4.2)	18	50	.403	.482
CMHI	10–50	30	35.6 (5.1)	18	50	.573	.632
MAKS Total	12–60	36	41.6 (5.9)	18	60	.653	.530
C1–C6	6–30	18	—	—	—	.720	—
C14–C19	6–30	18	—	—	—	.805	—
RIBS Total	4–20	12	13.1 (3.2)	4	20	.864	.807

CMHI, Community Mental Health Ideology; α, Cronbach’s alpha; ω, McDonald’s omega. RIBS total = items 5–8; items 1–4 are descriptive contact items.

Descriptive analysis of the four RIBS contact items (not included in the total score) indicated that 33.7% of participants reported having lived with someone with a mental or psychological illness, 34.1% had worked with such an individual, 28.6% reported having a neighbour with mental illness, and 31.9% had a close friend with mental illness. These findings suggest that approximately one third of participants had experienced some form of prior direct contact.

### 3.5 Bivariate associations

Age showed weak but statistically significant positive correlations with CAMI total (*r* = .086, *p* <.001) and all CAMI subscales (all *p*s <.05), and a weak negative correlation with RIBS (*r* = −.072, *p* = .005), indicating that younger participants reported somewhat greater behavioural willingness. Age was weakly positively correlated with MAKS (*r* = .065, *p* = .010).

No statistically significant sex differences were found for CAMI total score, any CAMI subscale, or MAKS (all *p*s >.05). However, males reported significantly higher RIBS scores than females (*M* = 13.9, *SD* = 3.4 vs. *M* = 13.0, *SD* = 3.2; *t*(1555) = 3.78, *p* <.001, *d* = 0.26), representing a small effect.

One-way ANOVAs revealed significant between-country differences for CAMI total (*F*(5, 1551) = 4.01, *p* = .001, partial η² = .013), Authoritarianism (*F* = 4.41, *p* <.001, partial η² = .014), Benevolence (*F* = 3.12, *p* = .008, partial η² = .010), Social Restrictiveness (*F* = 2.67, *p* = .021, partial η² = .009), MAKS (*F* = 4.36, *p* <.001, partial η² = .014), and RIBS (*F* = 3.49, *p* = .004, partial η² = .011). CMHI did not differ significantly across countries (*p* = .115). All partial η² values were small (<.015), indicating limited practical significance. Bonferroni-corrected *post-hoc* comparisons revealed that UAE participants reported significantly higher CAMI total (*d* = 0.51) and Authoritarianism (*d* = 0.57) scores than Kuwaiti participants, and Saudi participants reported higher MAKS scores than Kuwaiti participants (*d* = 0.31). No other pairwise comparisons survived Bonferroni correction.

Education was significantly associated with CAMI total, Authoritarianism, Benevolence, CMHI, and MAKS (all *p*s <.05, all partial η² <.01), but not with Social Restrictiveness or RIBS. Occupation was significantly associated with CAMI total, all CAMI subscales, and MAKS (all *p*s <.05, all partial η² ≤.02), but not with RIBS (*p* = .178). Participants in the health-related sector consistently reported the highest knowledge and most favourable attitudes (see **Table 3**).

**Table 3:** Bivariate associations between sociodemographic variables and study outcomes (N = 1, 557).

Panel A: Sex differences (independent-samples t-tests)												
Outcome	Male M (SD)			Female M (SD)		t		df		p	Cohen’s d	
CAMI Total	135.9 (16.7)			135.1 (16.2)		0.68		1555		.498	0.047	
Auth	32.8 (5.3)			32.6 (5.3)		0.63		1555		.531	0.043	
Bene	34.8 (5.6)			34.8 (5.4)		0.07		1555		.944	0.005	
SR	32.6 (4.2)			32.2 (4.2)		1.23		1555		.219	0.085	
CMHI	35.7 (5.0)			35.5 (5.1)		0.42		1555		.671	0.029	
MAKS	41.7 (6.3)			41.6 (5.9)		0.23		1555		.821	0.016	
RIBS	13.9 (3.4)			13.0 (3.2)		3.78		1555		<.001	0.262	
Panel B: Country differences (one-way ANOVA with bonferroni *post-hoc*)												
Outcome	Kuwait M (SD) n=1254	KSA M (SD) n=179		Bahrain M (SD) n=47	Qatar M (SD) n=20	Oman M (SD) n=17		UAE M (SD) n=40	F	p	η²	*Post-hoc*
CAMI Total	134.5 (15.9)	136.1 (16.5)		140.5 (15.4)	142.3 (19.1)	135.5 (18.7)		142.7 (20.8)	4.01	.001	.013	UAE>KW*
Auth	32.4 (5.2)	32.9 (5.8)		34.1 (4.7)	35.0 (5.8)	31.9 (5.9)		35.4 (6.2)	4.41	<.001	.014	UAE>KW**
Bene	34.6 (5.3)	35.6 (5.8)		36.3 (5.3)	36.2 (5.6)	34.5 (7.2)		36.6 (6.5)	3.12	.008	.010	—
SR	32.2 (4.1)	32.0 (4.3)		33.4 (4.4)	33.5 (4.3)	33.2 (4.2)		33.8 (5.1)	2.67	.021	.009	—
CMHI	35.4 (5.1)	35.5 (4.5)		36.6 (4.8)	37.5 (6.2)	35.8 (5.9)		37.0 (5.8)	1.77	.115	.006	—
MAKS	41.3 (5.9)	43.1 (6.0)		42.5 (6.3)	41.6 (5.6)	43.5 (7.4)		43.3 (4.6)	4.36	<.001	.014	KSA>KW**
RIBS	13.1 (3.1)	12.6 (3.5)		13.8 (3.3)	13.8 (1.5)	15.1 (4.2)		13.9 (3.9)	3.49	.004	.011	—
Panel C: Education differences (one-way ANOVA)												
Outcome	Primary n=3	Middle n=44			High school n=197		Undergrad n=1230		Postgrad n=83	F	p	η²
CAMI Total	113.0 (6.2)	129.7 (14.5)			136.2 (16.7)		135.3 (16.1)		135.6 (17.3)	2.89	.021	.007
Auth	24.3 (2.1)	30.6 (5.2)			32.8 (5.1)		32.6 (5.3)		33.0 (6.0)	3.47	.008	.009
Bene	29.3 (2.9)	32.3 (5.0)			34.7 (5.5)		35.0 (5.4)		34.6 (5.8)	3.43	.008	.009
SR	29.3 (4.2)	33.0 (3.0)			32.5 (4.6)		32.2 (4.1)		32.5 (4.6)	0.99	.412	.003
CMHI	30.0 (7.2)	33.9 (5.1)			36.3 (5.3)		35.5 (5.0)		35.6 (4.7)	3.12	.014	.008
MAKS	37.3 (2.3)	39.6 (4.9)			40.9 (5.7)		41.8 (5.9)		41.7 (6.8)	2.70	.029	.007
RIBS	16.7 (5.8)	13.6 (3.0)			13.3 (3.0)		13.1 (3.2)		13.5 (3.6)	1.61	.170	.004
Panel D: Occupation differences (one-way ANOVA)												
Outcome	Employee n=392		Student n=930	Unemp. n=92	Retired n=70	Self-emp. n=37	Unable n=10		Health n=26	F	p	η²
CAMI Total	137.1 (17.1)		133.9 (15.8)	139.1 (15.7)	134.3 (12.9)	132.5 (17.2)	138.8 (15.0)		144.5 (19.8)	4.54	<.001	.017
Auth	33.0 (5.5)		32.3 (5.3)	33.5 (4.9)	32.0 (4.5)	33.0 (5.9)	34.1 (3.8)		35.4 (6.5)	2.86	.009	.011
Bene	35.3 (5.2)		34.5 (5.6)	36.4 (5.6)	34.4 (4.4)	33.3 (5.2)	35.3 (5.3)		37.7 (5.7)	4.35	<.001	.017
SR	32.6 (4.6)		32.0 (4.0)	32.9 (3.8)	32.1 (3.5)	31.6 (4.9)	33.1 (3.8)		34.0 (5.5)	2.43	.024	.009
CMHI	36.2 (5.3)		35.2 (5.0)	36.4 (5.1)	35.8 (4.4)	34.5 (5.4)	36.3 (5.3)		37.4 (5.1)	3.09	.005	.012
MAKS	42.3 (5.9)		41.1 (5.9)	42.6 (5.8)	42.7 (6.2)	39.1 (6.5)	43.6 (5.6)		44.3 (6.0)	5.07	<.001	.019
RIBS	13.0 (3.3)		13.3 (3.2)	13.0 (3.3)	12.2 (2.8)	13.5 (3.1)	13.9 (2.5)		13.3 (3.7)	1.49	.178	.006
Panel E: Age correlations (pearson r)												
Variable Pair				r					p			
Age – CAMI Total				.086					<.001			
Age – Auth				.062					.015			
Age – Bene				.061					.017			
Age – SR				.068					.008			
Age – CMHI				.089					<.001			
Age – MAKS				.065					.010			
Age – RIBS				−.072					.005			

Auth, Authoritarianism; Bene, Benevolence; SR, Social Restrictiveness; CMHI, Community Mental Health Ideology; MAKS, Mental Health Knowledge Schedule; RIBS, Reported and Intended Behaviour Scale. η², partial eta-squared. Post-hoc comparisons used Bonferroni correction (15 pairwise comparisons). KW, Kuwait; KSA, Saudi Arabia. *p <.05; **p <.01. Higher CAMI/subscale scores indicate less stigmatizing attitudes. Higher MAKS scores indicate greater knowledge. Higher RIBS scores indicate greater behavioural willingness.

### 3.6 Correlations among knowledge, attitudes, and behavioural willingness

**Figure 1:**
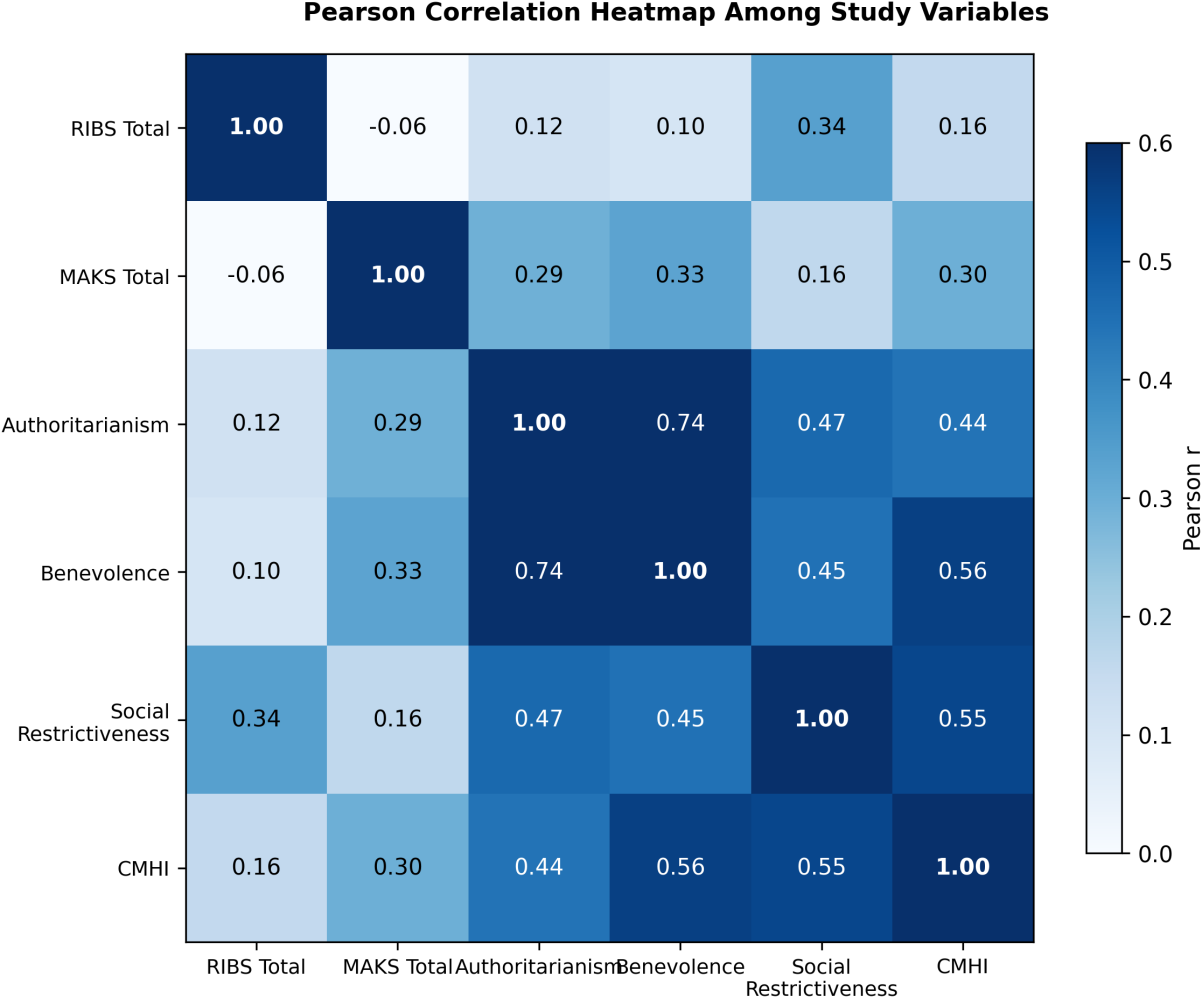
Heatmap illustrating Pearson correlation coefficients among behavioural engagement toward individuals with mental illness (RIBS total), mental health knowledge (MAKS total), and Community Attitudes toward Mental Illness (CAMI) subscales. Darker blue shades represent stronger positive correlations.

CAMI total showed a moderate positive correlation with MAKS (*r* = .341, *p* <.001), indicating that greater knowledge was associated with more favourable attitudes. The association between CAMI and RIBS was weaker but significant (*r* = .209, *p* <.001), suggesting that more positive attitudes were associated with more inclusive behavioural intentions, although this translation was modest. At the subscale level, MAKS was moderately correlated with Benevolence (*r* = .328), CMHI (*r* = .297), and Authoritarianism (*r* = .294), and weakly correlated with Social Restrictiveness (*r* = .161; all *p*s <.001). RIBS showed its strongest subscale association with Social Restrictiveness (*r* = .336) and weaker associations with CMHI (*r* = .156), Authoritarianism (*r* = .119), and Benevolence (*r* = .102; all *p*s <.001). Notably, the zero-order correlation between MAKS and RIBS was weak and negative (*r* = −.059, *p* = .020), suggesting that knowledge and behavioural willingness were not directly linked at the bivariate level. Full correlations are reported in **Table 4** and **Figure 1**.

**Table 4:** Pearson correlations among CAMI, MAKS, RIBS, and CAMI subscales.

Variable pair	r	p
CAMI total – MAKS	.341	<.001
CAMI total – RIBS	.209	<.001
MAKS – RIBS	−.059	.020
MAKS – Authoritarianism	.294	<.001
MAKS – Benevolence	.328	<.001
MAKS – Social Restrictiveness	.161	<.001
MAKS – CMHI	.297	<.001
RIBS – Authoritarianism	.119	<.001
RIBS – Benevolence	.102	<.001
RIBS – Social Restrictiveness	.336	<.001
RIBS – CMHI	.156	<.001
Age – CAMI total	.086	<.001
Age – MAKS	.065	.010
Age – RIBS	−.072	.005

### 3.7 Predictors of behavioural engagement

A simultaneous-entry multiple linear regression predicted RIBS total from the four CAMI subscales, MAKS total, age, sex, education level, country, and occupation (19 predictors total). Following data cleaning of implausible age values, the model was estimated using *N* = 1, 485. The model was statistically significant, *F*(19, 1465) = 13.27, *p* <.001, and explained 14.7% of the variance in behavioural engagement (*R*² = .147, adjusted *R*² = .136). This corresponds to a Cohen’s *f*² of 0.172, indicating a medium effect size (67), consistent with the typical magnitude of attitude-to-behaviour prediction models in stigma research (71).

Regression assumptions were assessed prior to analysis. The Durbin-Watson statistic was 1.93, indicating independence of residuals. Multicollinearity diagnostics indicated no evidence of problematic multicollinearity, with VIF values ranging from 1.02 to 1.68 and tolerance values ranging from 0.594 to 0.981 (66). Visual inspection of residual plots demonstrated acceptable linearity and homoscedasticity. Cook’s distance values indicated no excessively influential observations, with a maximum Cook’s distance of 0.214. The Q-Q plot demonstrated approximate normality of residuals, with only minor deviations at the distribution tails, considered acceptable given the large sample size (65).

**Figure 2:**
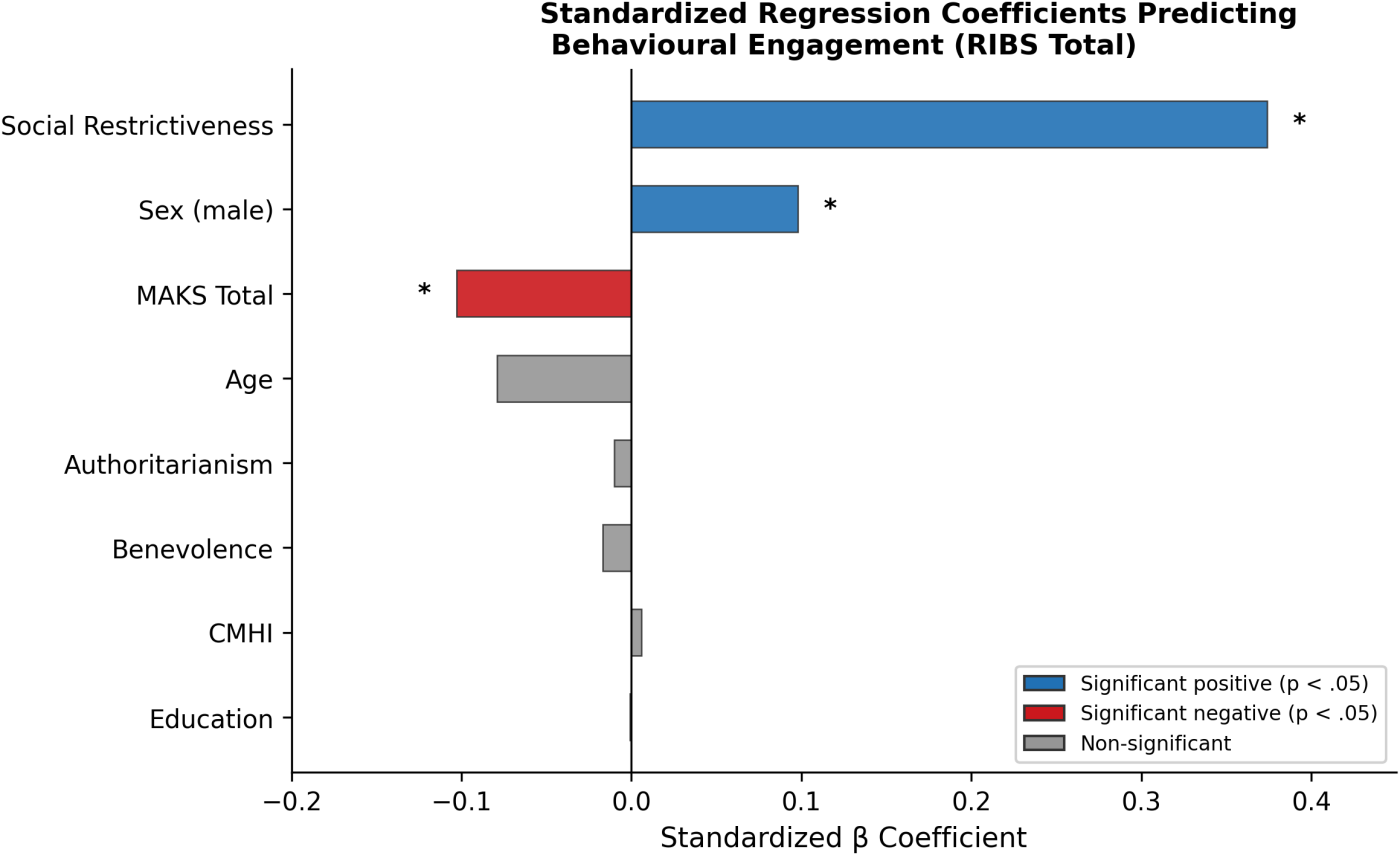
Standardized regression coefficients from the multiple linear regression model predicting behavioural engagement (RIBS total). Significant predictors are marked ***p < .001; non-significant predictors are shown in grey. Social Restrictiveness demonstrated the strongest positive association.

Social Restrictiveness was the strongest predictor (β = .374, *p* <.001), indicating that attitudes related to social distance had the greatest unique contribution to behavioural willingness. Given the low reliability of this subscale (α = .403) and the poor CFA fit, this finding should be regarded as exploratory; however, classical errors-in-variables theory indicates that the true association is likely underestimated due to attenuation from measurement error (69, 72). Sex was also significant (β = .098, *p* <.001), with males reporting higher intended behavioural engagement. MAKS total emerged as a significant negative predictor after controlling for attitudinal variables (β = −.103, *p* <.001), a finding consistent with a classical suppression effect (73, 74), as the zero-order MAKS–RIBS correlation was near zero (*r* = −.059). The VIF for MAKS was 1.19, ruling out multicollinearity as an explanation for this pattern. Age demonstrated a marginal negative association with behavioural engagement; however, this did not reach statistical significance following data cleaning (*B* = −0.079, *p* = .052). No other predictors—including Authoritarianism, Benevolence, CMHI, education, country, or occupation—reached statistical significance (see **Table 5** and **Figure 2**).

**Table 5:** Multiple linear regression predicting behavioural engagement (RIBS Total; N = 1, 485).

Predictor	β	p	VIF
Social Restrictiveness	.374	<.001	1.60
Sex (male = 1)	.098	<.001	1.06
MAKS total	−.103	<.001	1.19
Age	−.079	.052	2.67
Authoritarianism	−.010	.788	2.37
Benevolence	−.017	.661	2.65
CMHI	.006	.848	1.80
Education	−.001	.951	1.05
Country dummies	—	ns	< 1.68
Occupation dummies	—	ns	< 1.40

B, standardized coefficient; VIF, variance inflation factor. Model: F(19, 1465) = 13.27, p <.001, R² = .147, Adj. R² = .136. Durbin-Watson = 1.93. Max Cook’s D = 0.214. Cohen’s f² = 0.172.

A supplementary regression using CAMI total score as the sole attitudinal predictor (replacing the four subscale scores) confirmed the robustness of the primary pattern. CAMI total significantly predicted RIBS (β = .261, *B* = 0.051, *SE* = 0.005, 95% CI [0.041, 0.062], *p* <.001), and the MAKS suppression effect persisted (β = −.139, *p* <.001). Sex remained significant (β = .104, *p* <.001). The overall model explained 9.1% of the variance (*F*(16, 1527) = 9.52, *p* <.001).

### 3.8 Sensitivity analyses

Following reviewer feedback and additional quality screening, implausible age values were identified and treated as missing. The corrected age distribution demonstrated a mean of 26.65 years (*SD* = 10.53; range = 17–68). Regression analyses were subsequently recomputed using the cleaned dataset (*N* = 1, 485), producing improved model fit (*R*² = .147, up from.136 in the uncleaned dataset). All primary findings remained substantively identical: Social Restrictiveness remained the strongest predictor (β = .374, *p* <.001), sex remained significant, and the MAKS suppression effect persisted. Age, which had been marginally significant in the uncleaned dataset, no longer reached statistical significance (*p* = .052), suggesting that the original age finding may have been influenced by implausible data entries.

A further sensitivity analysis restricted to Kuwait-only respondents confirmed the same pattern, with Social Restrictiveness emerging as the strongest predictor of behavioural willingness. The consistency of findings across data-cleaning and subsample conditions supports the robustness of the primary results despite the sample composition limitations.

## 4 Discussion

This cross-sectional study of 1, 557 adults from six GCC countries found that public perspectives toward mental illness were mixed rather than uniformly positive or negative. Respondents demonstrated moderate mental-health knowledge and endorsed some support for rights-based and recovery-oriented principles, but they also showed substantial hesitation regarding public responsibility, community trust, and close social contact with people who have mental illness. This pattern is consistent with prior international evidence showing that abstract sympathy can coexist with social distance and exclusionary attitudes in everyday situations (2, 10, 12, 30), and extends these findings to an underrepresented Gulf Arab context.

### 4.1 The knowledge–attitude–behaviour relationship

The moderate positive correlation between MAKS and CAMI (*r* = .341) supports the conceptual link between MHL and stigma-related attitudes, consistent with theoretical models positing that knowledge shapes attitudes (1, 21). However, the weaker correlation between CAMI and RIBS (*r* = .209) underscores a recurring finding in stigma research: knowledge and favourable attitudes do not automatically translate into behavioural inclusion. This finding provides GCC-specific evidence for the well-established attitude–behaviour gap (12, 14, 26, 30). The implication for intervention design is clear: programmes that focus exclusively on increasing knowledge may yield attitudinal improvement without commensurate behavioural change. This reinforces recommendations from the Lancet Commission on stigma (3) that effective anti-stigma strategies must move beyond information provision to address the social and relational determinants of exclusionary behaviour.

### 4.2 Social restrictiveness as the dominant predictor

The finding that Social Restrictiveness was the strongest predictor of behavioural willingness (β = .374) warrants particular attention, albeit with important psychometric caveats. The low reliability of this subscale (α = .403, ω = .482) and the poor fit of the four-factor CFA model (CFI = .673, TLI = .653, RMSEA = .141, SRMR = .154) mean that the Social Restrictiveness construct, as measured by the original CAMI items, may not function as a stable or culturally equivalent latent dimension in this GCC Arabic sample. Crucially, however, classical psychometric theory dictates that measurement error attenuates observed regression coefficients toward zero (64, 69, 72). A significant predictor measured with poor reliability is therefore more rather than less notable: the disattenuated true effect would be considerably larger. Item-level analyses indicated that the reduced reliability was not attributed to a single problematic item but rather reflected modest inter-item correlations across several items, with no single item deletion substantially improving reliability. These findings underscore the need for Arabic-language CAMI item refinement—a recommendation supported by Sanabria-Mazo et al.’s (57) systematic review finding that CAMI subscale reliability is “not supported” across multiple international adaptations, with authoritarianism alphas as low as.27.

With this interpretive caution firmly in mind, the data suggest — tentatively — that reluctance to engage in close social proximity with people who have mental illness may be a more consequential dimension of public stigma in this sample than ignorance or absence of sympathy. In GCC societies, where family reputation (sharaf), social standing, and community perceptions of respectability carry substantial weight, social restrictiveness may plausibly be reinforced by concerns about how association with mental illness would reflect on one’s family or marriage prospects, although this cultural interpretation should be regarded as a hypothesis generated by the present data rather than a conclusion established by it (42–45, 47). This interpretation is consistent with attribution-based models of stigma, in which social distance is driven less by personal animosity than by perceived social costs and normative expectations (1, 6). It also aligns with findings from Pescosolido et al. (30), who found that in the United States, desire for social distance persisted despite improved public understanding of biological causation—suggesting that the mechanisms underlying social restrictiveness may be partially independent of knowledge and sympathy.

If confirmed in future research with psychometrically stronger Arabic social-restrictiveness measures, this pattern would suggest that anti-stigma interventions in the GCC should address not only individual knowledge and attitudes but also the social-evaluative concerns, family-level dynamics, and perceived reputational costs that sustain exclusionary behaviour. Structural interventions—such as anti-discrimination legislation, inclusive workplace policies, and public-facing recovery narratives—may be needed to shift the normative context in which social restrictiveness operates.

### 4.3 The MAKS suppression effect

The negative association between MAKS and RIBS in the regression model (β = −.103), despite a near-zero bivariate correlation (*r* = −.059), constitutes a classical suppression configuration as formally defined by Conger (73) and Tzelgov and Henik (74, *Psychological Bulletin*) and shown to be substantively replicable in personality research by Paulhus et al. (75, *Multivariate Behavioral Research*). In suppression, a predictor that is uncorrelated with the criterion increases the predictive validity of another predictor when entered jointly, by removing irrelevant variance. This phenomenon is distinct from multicollinearity—the MAKS VIF was only 1.19—and has been documented as interpretable rather than artifactual in a range of psychological research domains (76).

We offer one plausible, culturally grounded reading of this suppression pattern, consistent with — but not confirmed by — the present cross-sectional data. In collectivist Gulf Arab societies, recognizing that someone has a stigmatized mental illness activates a calculus of reputational risk operating through the cultural mechanisms of family honour (sharaf and ʿird), courtesy stigma (5, 77), and marriage-market penalties (42, 52, 53). A more knowledgeable individual may be better able to identify mental illness in others but simultaneously more aware of the social consequences of association—including diminished marriageability, reputational damage to the family unit, and community-level ostracism. Controlling for attitudes may therefore isolate a component of knowledge that functions as what might be termed strategic risk awareness: an understanding of mental illness that, in a context where disclosure and association carry tangible social costs, reduces rather than increases willingness for close interpersonal contact.

This interpretation is supported by converging evidence. Internationally, Schomerus et al. (12) found that biogenetic causal explanations sometimes increased perceptions of difference and social distance, and Doll et al. (78) reported that increased mental health literacy had not reduced stigmatization of depression and psychosis in a community sample. In the Arabic-language literature, Ben Amor et al. (58) documented in a Tunisian MAKS/RIBS validation (*n* = 2, 501) that “men performed better than women in terms of behavior toward people with mental illness, while women had a greater level of knowledge about mental health”—the identical knowledge↑/behaviour↓ pattern. Qualitative Gulf Arab research has documented that “concerns about family reputation, judgment from the community, and potential impacts on marriage prospects or social status” constitute primary barriers to mental-health engagement (53), and that Arab families “may refuse psychotherapy to protect the reputation of the family” even when they possess adequate clinical understanding (42).

This finding was not pre-registered and should be regarded as exploratory. The consistency of the suppression pattern across the full sample, the Kuwait-only subsample, the age ≥ 18 sensitivity analysis, and the CAMI-total regression model supports its robustness, but replication in independent GCC samples with alternative knowledge measures is needed before firm theoretical conclusions can be drawn. The sociocultural mechanism proposed here remains speculative and requires prospective, mixed-methods investigation; alternative statistical explanations, including coefficient instability due to shared measurement error among attitudinal predictors, cannot be fully excluded.

### 4.4 Prior contact and implications for contact-based interventions

Prior contact with people with mental illness, reported by approximately one third of participants, strengthens the conceptual rationale for contact-based interventions in the GCC. Although contact variables were not included in the regression model, the international literature consistently demonstrates that meaningful, positive contact reduces prejudice and social distance by challenging stereotypes and humanizing lived experience (3, 31, 33, 79). The relatively low prevalence of reported close contact in this sample suggests considerable room for structured contact-based programming. Future studies should directly test whether contact predicts attitudes and behavioural willingness in GCC populations and investigate how cultural norms about disclosure and social association moderate the contact–stigma relationship.

### 4.5 Demographic patterns

The finding that males reported significantly higher intended behavioural engagement than females (*d* = 0.26) warrants interpretation through the lens of gendered social norms specific to GCC collectivist societies. In Arab Gulf contexts, women are frequently positioned as the primary carriers of family honour (*sharaf*); their social conduct, relationships, and associations are subject to heightened familial and communal scrutiny (41, 42, 80). The social cost of associating with stigmatized groups—including people with mental illness—is therefore disproportionately higher for women, for whom such associations may be perceived as threatening to marriageability, family reputation, and community standing (52, 53).

This interpretation is empirically supported by converging regional evidence. Bener and Ghuloum (81) found in a Qatari sample that “most of the women were afraid and not willing to keep friendships with the mentally ill” despite comparable or higher knowledge levels. Ben Amor et al. (58) documented in a Tunisian Arabic sample that “men performed better than women in terms of behavior toward people with mental illness, while women had a greater level of knowledge about mental health.” The consistency of this gender pattern across Qatari, Tunisian, and Kuwaiti samples—with women showing equal or superior knowledge but lower behavioral willingness—suggests a robust cultural mechanism rather than a study-specific artifact.

**Figure 3:**
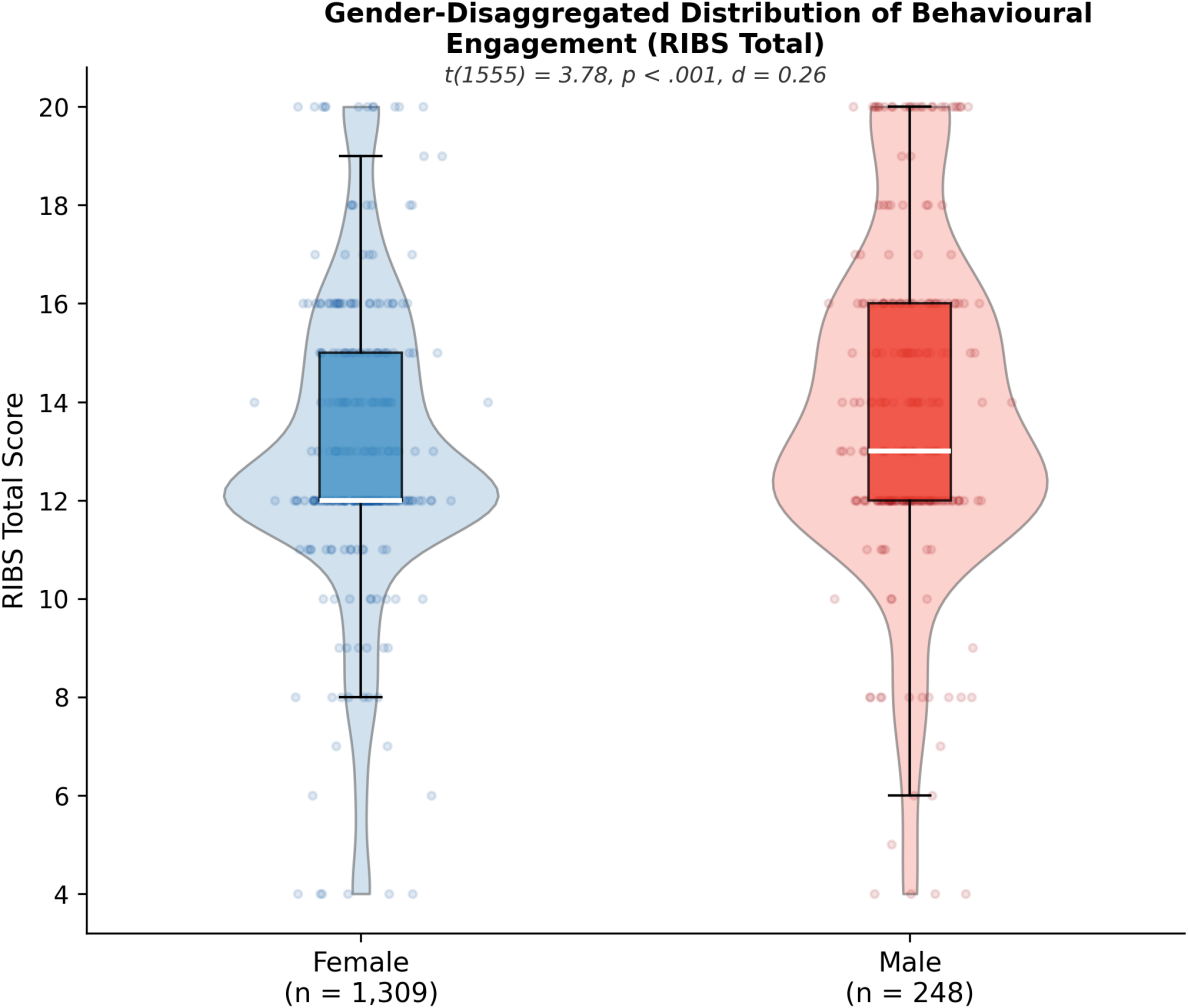
Gender-disaggregated distributions of behavioural engagement (RIBS total). Violin-boxplots with jittered observations illustrate score distribution, central tendency, and variability across male and female participants.

Furthermore, in GCC societies where gender segregation structures social interaction, men may perceive greater social latitude for contact with unfamiliar individuals in public and occupational settings, while women’s social interactions are more often mediated by family networks where reputational consequences are more immediate (45, 80). These findings suggest that anti-stigma interventions in the GCC should be gender-responsive, addressing the distinctive reputational pressures that women face and potentially engaging family-level stakeholders rather than targeting individual women in isolation (see **Figure 3** for gender-disaggregated distributions).

The age effect (younger participants reporting greater behavioural willingness) is consistent with international literature suggesting generational cohort differences in openness to mental-health discourse and reduced stigma (12, 26). However, the effect was small (β = −.081), and the cross-sectional design cannot disentangle age from cohort effects.

### 4.6 Psychometric considerations

The psychometric results are noteworthy for both their strengths and limitations. The CAMI total and RIBS scales performed well (α = .836/.864; ω = .816/.807), supporting their use in Arabic-language GCC research. However, CAMI subscale reliabilities ranged from α = .403 (Social Restrictiveness) to α = .651 (Authoritarianism), with McDonald’s ω values ranging from.482 to.677. This variability is consistent with the systematic review by Sanabria-Mazo et al. (57), which documented heterogeneous subscale performance across CAMI translations and populations. The CFA indicated poor fit of the original four-factor CAMI structure in this Arabic sample (CFI = .673, RMSEA = .141)—a pattern reported across multiple cultural adaptations. Future Arabic-language research should prioritize item refinement of the CAMI, and consider whether alternative or abbreviated social-restrictiveness scales may better serve GCC populations.

**Figure 4:**
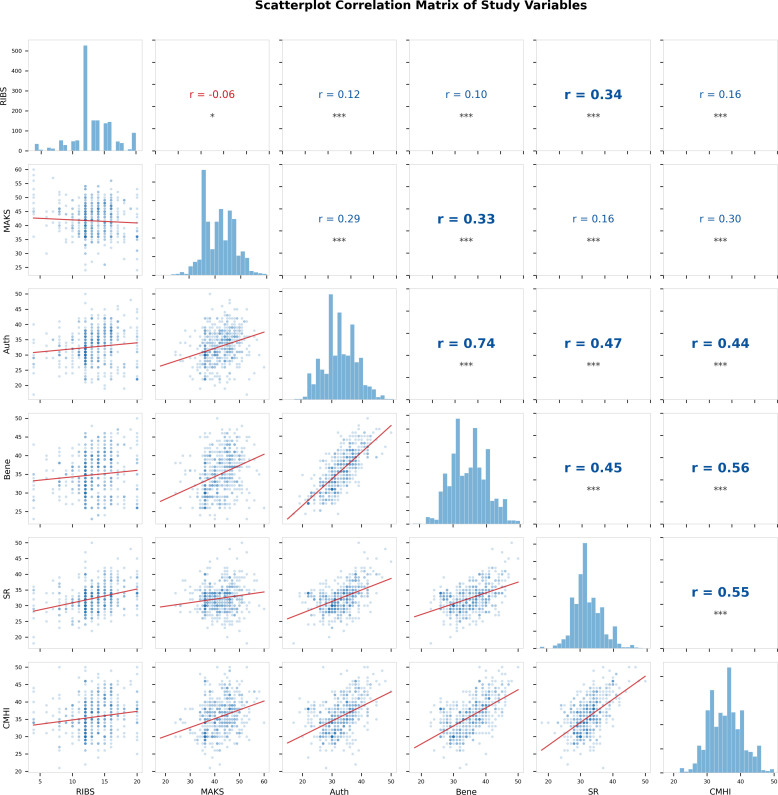
Scatterplot correlation matrix illustrating interrelationships among behavioural engagement (RIBS total), mental-health knowledge (MAKS total), and CAMI subscales. Pearson correlation coefficients are displayed in the upper triangle. ***p < .001; **p < .01; *p < .05.

The low reliability of Social Restrictiveness is particularly consequential because this subscale emerged as the study’s strongest predictor; however, as noted above, the true association may be underestimated due to measurement error, making the finding more rather than less notable. The MAKS total reliability (α = .653, ω = .530) fell below conventional thresholds, consistent with prior findings in Arabic adaptations (58). The component sections showed acceptable (C1–C6: α = .720) to good (C14–C19: α = .805) reliability when examined separately. The full pattern of inter-construct correlations underlying these psychometric observations is visualised in the scatterplot correlation matrix (**Figure 4**).

### 4.7 Implications for practice and policy

First, the emergence of Social Restrictiveness as the strongest predictor of behavioural willingness suggests that anti-stigma interventions should move beyond general awareness campaigns and directly target beliefs about social distance, everyday coexistence, and the perceived social costs of association with mental illness. This may include normalizing recovery narratives, showcasing people with mental illness in valued social roles, and addressing family-level concerns about reputation and marriageability. Second, the low prevalence of reported prior contact suggests a need for contact-based interventions creating structured, positive interactions between the public and people with lived experience of mental illness, adapted to GCC cultural norms—for example, through community events, media campaigns featuring local voices, or peer-led university workshops. Third, universities, youth settings, and social media represent promising intervention platforms given the demographic profile of the present sample. Fourth, the findings have implications for health-professional education; occupational therapists, who promote social participation, daily-life engagement, and community inclusion, are well positioned to implement and evaluate anti-stigma interventions that target the behavioural dimension of stigma (3). Finally, policymakers should consider structural-level interventions—including workplace anti-discrimination policies, media guidelines, and community mental-health service expansion—that address institutional dimensions of stigma alongside individual-level change.

### 4.8 Strengths and limitations

This study has several strengths, including a large sample (*N* = 1, 557), inclusion of respondents from all six GCC countries, and the simultaneous use of three established, internationally recognised stigma instruments. The use of MAKS, RIBS, and CAMI together enabled a multi-domain assessment of knowledge, attitudes, and behavioural willingness that is rare in existing GCC stigma research. The study also provides the first direct regression test of which stigma dimensions predict behavioural engagement in a GCC public sample. The comprehensive regression diagnostics, multiple sensitivity analyses, and psychometric transparency (including item-level analysis, EFA, and reporting of McDonald’s ω) strengthen confidence in the reported findings.

However, several limitations must be acknowledged. First, the cross-sectional design precludes causal inference; the observed associations among knowledge, attitudes, and behaviour may not reflect directional relationships. Second, the sample was non-probabilistic and heavily weighted toward Kuwait (80.5%), women (84.1%), and university students (59.7%), which limits generalizability to the broader GCC adult population. This profile is consistent with comparable Arabic-language online stigma surveys (58, *n* = 2, 501, 73% female students; 59, *n* = 9, 782, 77.1% female), suggesting it reflects the characteristics of the accessible population for online Arabic-language health research rather than a unique deficiency of the present study. The overrepresentation of young, educated women may have inflated knowledge and attitude scores. Third, small subsamples from several countries (e.g., *n* = 17 from Oman) constrain the reliability of between-country comparisons, and only two pairwise differences survived Bonferroni correction. Fourth, the online self-report format introduces potential for self-selection bias and social desirability bias, particularly in a collectivist cultural context where public endorsement of stigmatizing attitudes may be perceived as socially unacceptable (45). The discrepancy between relatively favourable CAMI scores and more modest RIBS scores may partly reflect differential susceptibility to social desirability across attitudinal versus behavioural measures. Fifth, CAMI subscale reliabilities were variable (α = .403–.651; ω = .482–.677), and MAKS total reliability (α = .653) was below conventional thresholds, limiting confidence in subscale-level and knowledge-related findings. The CFA indicated poor fit of the original four-factor CAMI structure in this Arabic sample (CFI = .673, RMSEA = .141), consistent with international findings (57). Sixth, the regression model explained a modest proportion of variance (*R*² = .147, Cohen’s *f*² = 0.172), suggesting that important determinants of behavioural willingness—such as religiosity, family attitudes, perceived social norms, personal experience with mental illness, and media exposure—were not captured. Seventh, initial data inspection revealed implausible age entries likely reflecting typographical errors in the self-reported age field; these were treated as missing and regression analyses recomputed, with all primary findings confirmed. Future studies should implement real-time age-verification screening. Eighth, the Arabic versions of all three instruments were adopted from a published Lebanese study (40) without further cultural adaptation for Gulf Arabic dialect or independent psychometric validation in a GCC sample prior to use. The lower subscale reliabilities observed in the present study compared to the Lebanese source (e.g., SR α = .397 vs.690) suggest that some items may function differently across Arabic-speaking populations, and future research should conduct formal cross-cultural validation of these instruments in GCC contexts. Future research should employ probability-based sampling, balanced gender and national distributions, mixed-methods designs capable of capturing cultural mechanisms, and longitudinal approaches to examine causal pathways.

## 5 Conclusion

This cross-sectional study of adults from six GCC countries found that mental-health knowledge, stigma-related attitudes, and intended social behaviour are related but distinct domains, consistent with the social-cognitive model of stigma. Respondents often endorsed sympathetic and recovery-oriented principles yet showed substantial hesitation regarding close social contact with people who have mental illness. Greater knowledge was associated with more favourable attitudes, but the translation of attitudes into behavioural willingness was more limited, replicating the established attitude–behaviour gap in a Gulf Arab context. Social Restrictiveness emerged as the most powerful predictor of behavioural engagement—a finding that proved robust across data cleaning and sensitivity analyses, and that was confirmed by the more reliable CAMI total-score model—suggesting that social-evaluative concerns, rather than knowledge deficits or absence of sympathy, may constitute an important barrier to behavioural inclusion. A confirmatory factor analysis revealed poor fit of the original four-factor CAMI structure (CFI = .673, RMSEA = .141), underscoring the need for Arabic-language item refinement while noting that the significant Social Restrictiveness finding despite poor measurement may represent a conservative estimate of the true effect, pending confirmation with psychometrically stronger measures. A classical suppression effect for MAKS, tentatively interpreted through the lens of courtesy stigma and family-honour norms specific to Gulf Arab societies, raises the hypothesis that knowledge may paradoxically reduce intended contact by heightening awareness of social costs — a finding that warrants further investigation in GCC populations. These findings provide an important regional baseline and support the development of culturally grounded, gender-responsive, contact-based anti-stigma interventions that address not only what people know and believe about mental illness, but also the social norms, family dynamics, and reputational concerns that sustain exclusionary behaviour.

## Data Availability

The raw data supporting the conclusions of this article will be made available by the authors, without undue reservation.

## Supplementary material

Supplementary Table 1 Corrected item-total correlations for the 10 Social Restrictiveness items of the Arabic CAMI (N = 1,557).
