# Supplementary material for: Public knowledge, stigma, and social acceptance toward mental illness in the Gulf region: a cross-sectional survey
Source: Front Psychiatry. 2026 Jun 30;17:1860027. doi: 10.3389/fpsyt.2026.1860027 (PMC13364959; doi:10.3389/fpsyt.2026.1860027)
Supplement: Supplementary Table 1 — Corrected item-total correlations for the 10 Social Restrictiveness items of the Arabic CAMI (N = 1,557). [file Table1.docx]

**Supplementary Table S1**

*Corrected Item-Total Correlations for the Social Restrictiveness Subscale (N = 1,557)*

| **CAMI Item** | **Corrected Item-Total Correlation (r_it)** |
| --- | --- |
| Item 3 | .119 |
| Item 7 | .335 |
| Item 11 | −.056 |
| Item 15 | .299 |
| Item 19 | −.022 |
| Item 23 | .187 |
| Item 27 | −.013 |
| Item 31 | .320 |
| Item 35 | .296 |
| Item 39 | .147 |

***Note.*** *Items 11, 19, and 27 showed near-zero or negative corrected item-total correlations. No single item deletion substantially improved subscale reliability (α = .403; ω = .482), indicating broad measurement instability rather than a single problematic item.*
